# Fibroblast Growth Factor 23 and Cardiovascular Risk in Diabetes Patients—Cardiologists Be Aware

**DOI:** 10.3390/metabo12060498

**Published:** 2022-05-30

**Authors:** Anna Kurpas, Karolina Supel, Paulina Wieczorkiewicz, Joanna Bodalska Duleba, Marzenna Zielinska

**Affiliations:** 1 Department of Interventional Cardiology, Medical University of Lodz, 92-213 Lodz, Poland; a.lipinska.a@gmail.com (A.K.); karolina.supel@umed.lodz.pl (K.S.); paulina.wieczorkiewicz@umed.lodz.pl (P.W.); 2 Diabetes Outpatient Clinic ‘Poradnia Nowa’, 90-631 Lodz, Poland; j.bodalska.duleba@gmail.com

**Keywords:** fibroblast growth factor 23, ischemic heart disease, diabetes mellitus

## Abstract

Numerous clinical studies have indicated that elevated FGF23 (fibroblast growth factor 23) levels may be associated with cardiovascular (CV) mortality, especially in patients with chronic kidney disease. The purpose of this study was to examine the hypothesis that FGF23 may be a potent CV risk factor among patients with long-standing type 2 diabetes mellitus (T2DM). Research was performed utilizing patients with T2DM and regular outpatient follow-up care. Baseline characteristics determined by laboratory tests were recorded. Serum FGF23 levels were detected using a sandwich enzyme-linked immunosorbent assay. All patients underwent echocardiograms and 12-lead electrocardiograms. Data records of 102 patients (males: 57%) with a median age of 69 years (interquartile range (IQR) 66.0–74.0) were analyzed. Baseline characteristics indicated that one-third (33%) of patients suffered from ischemic heart disease (IHD), and the median time elapsed since diagnosis with T2DM was 19 years (IQR 14.0–25.0). The hemoglobin A1c, estimated glomerular filtration rate, and FGF23 values were, respectively, as follows: 6.85% (IQR 6.5–7.7), 80 mL/min/1.73 m^2^ (IQR 70.0–94.0), and 253.0 pg/mL (IQR 218.0–531.0). The study revealed that FGF23 was elevated in all patients, regardless of IHD status. Thus, the role of FGF23 as a CV risk factor should not be overestimated among patients with T2DM and good glycemic control.

## 1. Introduction

Fibroblast growth factor 23 (FGF23) is a 32 kDa glycoprotein with an N-terminal and a C-terminal region with 251 amino acids [1]. It belongs to a subfamily of endocrine fibroblast growth factors (FGF) and is secreted predominantly by osteoblasts and osteocytes in response to increased serum phosphate or 1,25-dihydroxyvitamin D [1,25(OH)_2_D]. FGF23 works as a phosphate-regulating hormone whose actions are mediated by the FGF receptors and coreceptor klotho. FGF23 primarily targets the kidney to enhance renal phosphate excretion and to suppress synthesis of 1,25(OH)_2_D by decreasing renal tubular 1α-hydroxylase expression. FGF23 also acts directly on the parathyroid gland, causing a decrease in parathyroid hormone (PTH) secretion.

The discovery of FGF23 has had a critical clinical impact [2]. It revolutionized not only the understanding of phosphate and vitamin D metabolism, but also of endocrine feedback loops among the parathyroid gland, intestines, bone, and kidney in order to maintain mineral homeostasis. Moreover, this finding resulted in: (1) development of a new classification of hereditary and acquired hypo- and hyperphosphatemic diseases [3,4]; (2) implementation of novel treatment approaches [5,6,7]; and (3) better understanding of disordered mineral metabolism in chronic kidney disease (CKD) and links between metabolic derangements and cardiovascular (CV) mortality. Medical investigations of FGF23 reflect the power of science to fulfill the unmet needs of patients diagnosed with rare diseases and reveal the basic pathophysiology of common diseases.

In recent years, FGF23 was widely discussed as a promising biomarker for early diagnosis of adverse outcomes and disease progression in patients with CKD [8,9]. Elevated FGF23 secretion precedes the increase in PTH and phosphate when eGFR decreases. It is generally known that FGF23 is also associated with increased CV morbidity and all-cause mortality in patients with CKD. However, various medical studies indicated that FGF23 is also highly related to cardiac complications, even independently of comorbidities. Emerging data imply a role for disturbances in phosphate metabolism in diabetes mellitus (DM) [10]. Supposedly, metabolites, such as phosphate and glucose, play an intricate game in DM, predisposing one to vascular calcification [11,12]. The question therefore arises regarding whether FGF23 elevation is directly linked to glucose metabolism.

Cardiovascular diseases (CVD) are consistently the leading cause of death worldwide [13]. Therefore, scientists are actively working to identify a novel CVD biomarker. Moreover, CVD is a major cause of mortality in the growing diabetic population [14]. The literature lacks evidence of an association between FGF23 and ischemic heart disease (IHD). In addition, there are barely any data on the relationship between FGF23 and glucose disturbances.

The key objective of this study was to determine whether FGF23 may be used as a CV risk factor among patients with long-standing type 2 diabetes mellitus (T2DM). Our study also aimed to assess the relationship between FGF23 and fasting glucose level. It is possible that FGF23 may become a predictive tool for this group of patients. Furthermore, we believe that it may offer a real chance to improve the survival rate of patients suffering from T2DM.

## 2. Results

**Figure 1:**
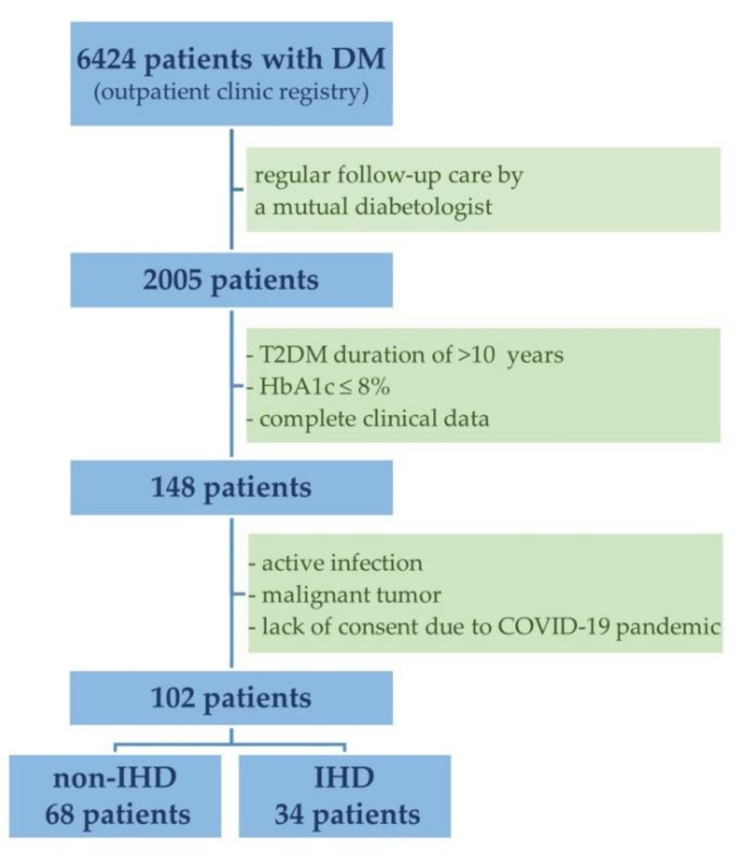
Flow chart for patient enrollment. Abbreviations: DM, diabetes mellitus; T2DM, type 2 diabetes mellitus; HbA1c, hemoglobin A1c; COVID-19, coronavirus disease 2019; IHD, ischemic heart disease.

After considering the inclusion criteria, the total study population of 102 patients comprised 68 subjects without IHD (Group 1) and 34 subjects with IHD (Group 2) (Figure 1).

Data from all individuals were analyzed. Our research included 43 women (42%) and 59 men (58%) with a median age of 69 years (interquartile range (IQR) 66.0–74.0). Baseline characteristics indicated that one-third (33%) of the patients suffered from IHD, and the median time elapsed since diagnosis with T2DM was 19 years (IQR 14.0–25.0). Patients had a prior history of arterial hypertension (HA) (86%), myocardial infarction (MI) (28%), coronary artery bypass graft surgery (CABG) (9%), atrial fibrillation (11%), and stroke (9%). We also explored the presence of chronic complications of DM, such as retinopathy (20%), neuropathy (12%), and diabetic foot syndrome (5%). The values of fasting and postprandial blood glucose levels were 120 mg/dL (IQR 110–130) and 150 mg/dL (IQR 130–170), respectively. We found no correlations between FGF23 and either fasting or postprandial blood glucose levels (r = −0.0608, *p* = 0.5499; r = −0.1512, *p* = 0.1573). Furthermore, 74% of patients reported smoking history (current and former smokers collectively). In our study population, 34% of subjects were classified as overweight and 45% obese. The presence of family history of heart disease and DM were 50% and 63%, respectively. Participants’ demographic and clinical data are presented according to IHD status in Table 1. None of these variables correlated with the FGF23 level. As shown, patients in Group 2 were more often men, had a prior history of MI and CABG, and less frequently were diagnosed with HA.

Our study also evaluated the concentration of FGF23, renal function, hemoglobin A1c (HbA1c), and a lipid profile. Table 2 presents the laboratory test results according to IHD status.

**Figure 2:**
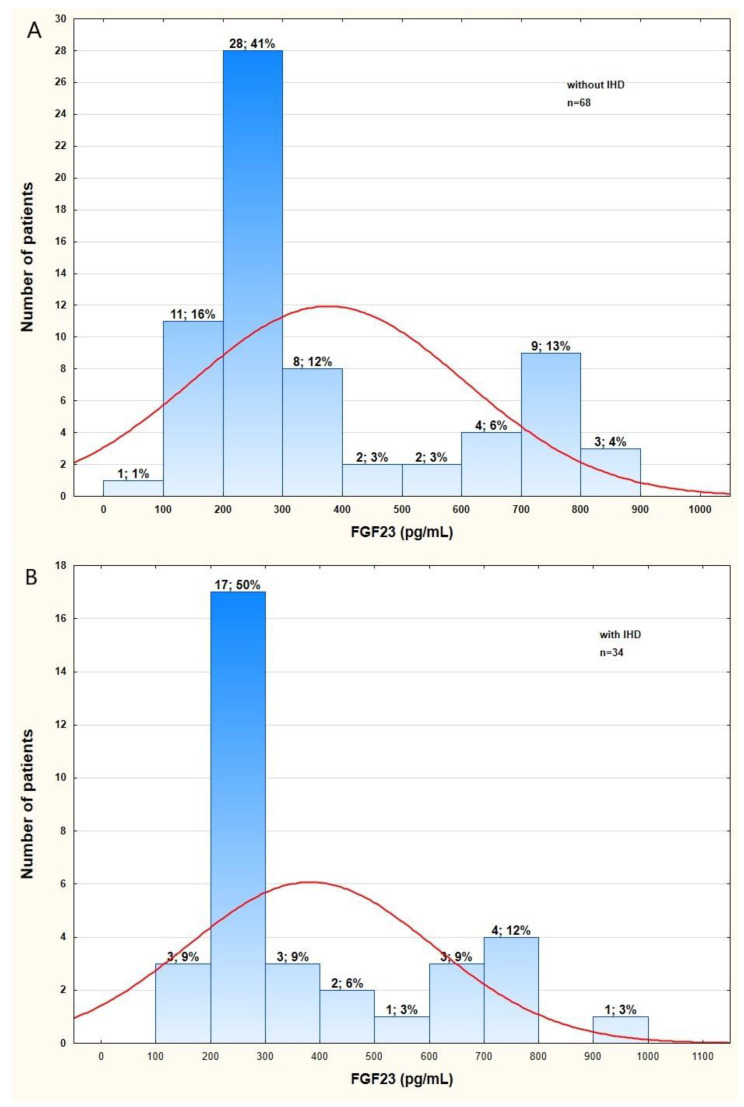
The distribution of FGF23 levels in patients with long-standing type 2 diabetes mellitus according to IHD status (**A**,**B**). Abbreviations: FGF23, fibroblast growth factor 23; IHD, ischemic heart disease.

FGF23 was elevated in all patients (253.0 pg/mL, IQR 218.0–531.0); however, the variable was not normally distributed (Figure 2). The predominant value of FGF23 varied from 200 to 300 pg/mL.

**Figure 3:**
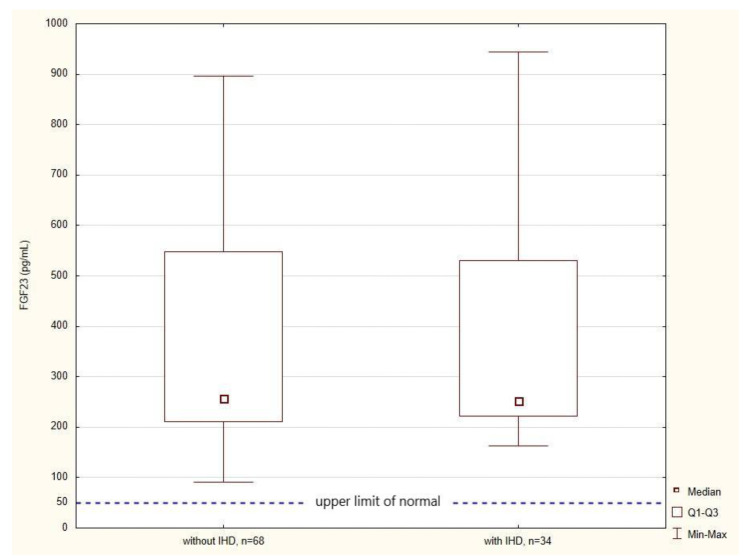
Box plots showing the median levels of FGF23 according to IHD status with reference to the upper limit of normal for FGF23 according to Yamazaki et al. [15]. Abbreviations: IHD, ischemic heart disease; FGF23, fibroblast growth factor 23.

Contrary to our expectations, there was no significant difference in FGF23 levels between Group 1 and Group 2 (258.0 pg/mL, IQR 212.0–548.0; 253.0 pg/mL, IQR 222.0–531.0), as shown in Figure 3.

**Figure 4:**
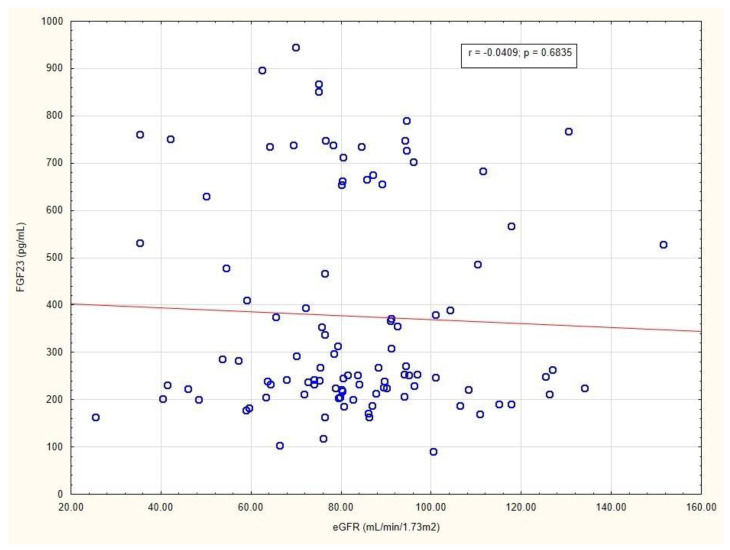
Correlation between eGFR and FGF23 levels in patients with long-standing type 2 diabetes mellitus. Abbreviations: eGFR, estimated glomerular filtration; FGF23, fibroblast growth factor 23.

The values of HbA1c, creatinine (Cr), and eGFR were, respectively, as follows: 6.85% (IQR 6.5–7.7), 0.86 mg/dL (IQR 0.75–1.0), and 80 mL/min/1.73 m^2^ (IQR 70.0–94.0). These results indicated that the study population comprised mainly patients with preserved renal function and good glycemic control. We found no correlation between FGF23 and HbA1c levels (r= −0.1562; *p* = 0.1170). Interestingly, there also was no correlation between eGFR and FGF23 levels (Figure 4).

A lipid profile consisted of the following tests: total cholesterol (TC) (152.0 mg/dL, IQR 125.0–186.0), high-density lipoprotein cholesterol (HDL-C) (49.0 mg/dL, IQR 42.0–59.0), low-density lipoprotein cholesterol (LDL-C) (72.0 mg/dL, IQR 51.0–98.0), non-high-density lipoprotein cholesterol (non-HDL-C) (96.0 mg/dL, IQR 75.0–126.0), and triglycerides (TG) (126.0 mg/dL, IQR 94.0–169.0). None of the laboratory variables correlated with FGF23 levels. Patients in Group 2 had a significantly higher level of Cr (*p =* 0.002) and a lower concentration of HDL-C (*p* = 0.029).

**Figure 5:**
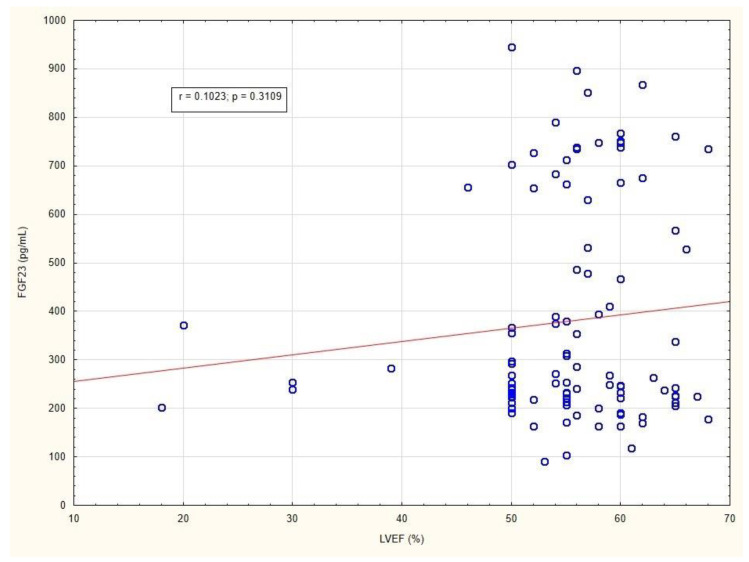
Correlation between LVEF and FGF23 level in patients with long-standing type 2 diabetes mellitus. Abbreviations: LVEF, left ventricular ejection fraction; FGF23, fibroblast growth factor 23.

All patients underwent full transthoracic echocardiography. The median left ventricular ejection fraction (LVEF) was 56% (IQR 52–60). No correlation was found between LVEF and FGF23 levels in patients (Figure 5).

As shown in Table 3, patients with overt IHD had higher values of the following parameters: left ventricular end systolic volume (LVESV) (*p =* 0.007), left ventricular end diastolic volume (LVEDV) (*p =* 0.009), left ventricular mass index (LVMI) (*p =* 0.004), left atrium (LA) volume (*p =* 0.0002), left atrial volume index (LAVI) (*p =* 0.0003), and tricuspid annular plane systolic excursion (TAPSE) (*p =* 0.008).

**Figure 6:**
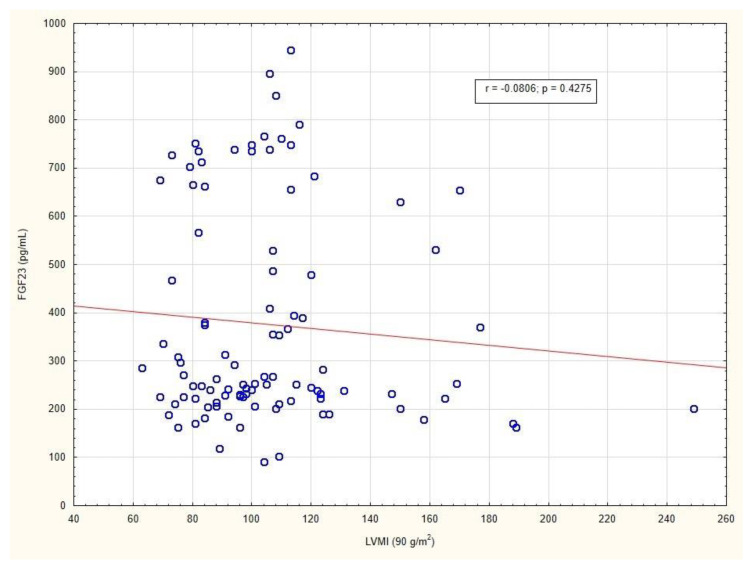
Correlation between LVMI and FGF23 level in patients with long-standing type 2 diabetes mellitus. Abbreviations: LVMI, left ventricular mass index; FGF23, fibroblast growth factor 23.

No significant difference was found in the right ventricular outflow tract, interventricular septum thickness at end-systole, or interventricular septum thickness at end-diastole between Group 1 and Group 2. Our data showed no evidence for association between FGF23 levels and any of the echocardiographic variables. Figure 6 presents the absence of a relationship between the LVMI and FGF23 levels in patients.

Table 4 gathers data on diabetes medications prescribed by a diabetologist. According to our results, none of the medication groups were associated with the FGF23 level.

## 3. Discussion

Little is known about the correlation between FGF23 and IHD, not to mention the relationship between FGF23 and DM. The overwhelming majority of these data refer to patients already diagnosed with a kidney disease, which is widely known as an initial trigger to the elevation of FGF23. Still, participants enrolled in our study had in most cases preserved renal function and good glycemic control despite a long-standing T2DM. Therefore, the interference of confounding factors on the FGF23 level was reduced to a certain extent. The main objective of this research was to determine whether FGF23 may be regarded as a CV risk factor among a diabetic population. Our in-depth analysis proved that FGF23 level was elevated in all patients; however, this was regardless of IHD status. Hence, the role of FGF23 as a potent CV risk factor should not be overestimated among these patients. Interestingly, the present study showed no evidence regarding associations between FGF23 and any of the variables, among them eGFR, HbA1c, fasting, and postprandial blood glucose levels. Diabetes medications appeared not to be related to the FGF23 level as well.

The term IHD encompasses a group of clinical syndromes resulting from myocardial ischemia with subsequent heart damage [16]. Coronary blood flow reduction is predominantly triggered by coronary artery atherosclerosis. Furthermore, IHD was recognized as the most frequent cause of HF. Its incidence and mortality rates are consistently rising [17]. IHD has been acknowledged as a leading cause of death and a public health problem worldwide [18]. Several studies demonstrated that IHD is a preventable and even eradicable disease, if only the risk factors are determined early and carefully controlled [17,19]. The most important risk factors for IHD were classified into two groups [20,21,22]. Age over 65 years, male sex, family history of heart disease, and ethnic background are considered the nonmodifiable risk factors, whereas HA, hyperlipidemia, DM, obesity, smoking, poor diet, and sedentary lifestyle belong to the group of modifiable risk factors. Our results were consistent with previous literature that referred to HA; however, they indicated that female rather than male sex should be regarded as a risk factor for IHD. Moreover, this study further added several variables that may be useful during risk stratification in IHD: Cr, eGFR, HDL-C, LVESV, LVEDV, LVMI, LA volume, LAVI, and TAPSE (*p* < 0.05). Identification of appropriate risk factors is essential for establishing an individual treatment plan and effective primary prevention of IHD. We must bear in mind that there is also, of no less importance, a primordial prevention defined as avoidance of the development of risk factors [23].

FGF23 was originally identified in 2000 through a gene mutation in patients with hypophosphatemic rickets and osteomalacia that causes an elevation in the FGF23 level [24]. The discovery of FGF23 has revolutionized, firstly, the understating of phosphate and vitamin D metabolism. Secondly, it led to development of a novel therapy of X-linked hypophosphatemia with the use of a monoclonal blocking antibody to FGF23 (burosumab) [5]. Beyond these rare diseases, an increase in FGF23 concentration is the first detectable change in mineral metabolism in CKD-mineral and bone disorder [9]. FGF23, as an endocrine-acting phosphaturic hormone, steadily rises with a decline in kidney function. Its intensive secretion seems to be crucial to avoid phosphate overload in CKD. Mounting evidence has confirmed a close association between an elevated FGF23 level and cardiac events and all-cause mortality in patients with CKD [8,25,26]. Surprisingly, we did not observe a significant correlation between FGF23 and eGFR, irrespective of IHD status. This may be explained by the fact that the median eGFR in the study population was 80 mL/min/1.73 m^2^ (IQR 70–94).

In the last decade, the role of FGF23 as a CV risk factor has become a subject of intense discussion. After thorough analysis of the literature, we concluded that the great majority of publications promoted FGF23 as a promising biomarker of CV risk. Kestenbaum et al. evaluated 6547 patients who were initially free of CVD, and followed up with them for 7.5 years [27]. It finally appeared that an increased level of FGF23 was associated with subclinical cardiac disease, new heart failure (HF), and a 14% greater risk of IHD. Coherent results were obtained by Udell and colleagues, who showed that the FGF23 level was independently associated with a higher risk of CV death or HF among patients with stable IHD [28]. Another large study showed that FGF23 concentration, measured among subjects undergoing coronary angiography, correlated significantly with risk for all-cause and CV mortality [29]. Numerous studies have shown a clear association between FGF23 level and CV morbidity and mortality in patients with HF rather than MI. German researchers conducted a study that indicated that FGF23 was linked to one-year mortality in patients with MI and concomitant acute HF [30]. Thorsen et al. demonstrated that during the acute phase of MI, the level of FGF23 began to decline, with its normalization at seven days following revascularization. Interestingly, during a 1-year follow-up, they observed a gradual rise in FGF23 concentrations in patients with overt HF [31]. According to Shibata and his collaborators, FGF23 was related to LVEF and LVMI independently of renal function and other calcium phosphate metabolism-related parameters [32]. Our study did not reveal an association between overt IHD (33% of study population) and the FGF23 level. Apparently, these results were consistent with recent findings. This may be explained by the fact that 88% of the subjects with IHD were assigned to Group 2 due to a prior history of MI. Furthermore, we found no correlation between the FGF23 level and basic echocardiographic parameters; e.g., LVEF. This most likely was due to the fact that the vast majority of patients had a preserved ejection fraction (56%, IQR 52–60) and did not suffer from HF, which is a predominant stimulus that induces FGF23 secretion.

DM is an insidious disease and has been acknowledged to be a silent killer. According to the World Health Organization, the diabetic population comprises about 422 million people worldwide. Each year, 1.5 million of them die due to complications directly attributed to DM. Adults with DM have a 2- to 3-fold increased risk of MI and stroke [14]. Furthermore, DM is a leading cause of CKD. Emerging data suggest that FGF23 may be elevated in patients with T2DM and may be linked to unfavorable outcomes, regardless of CKD. It is widely known that patients with T2DM are more susceptible to fractures and have a higher bone mineral density compared to patients without DM [33,34,35]. Recent studies even have demonstrated the association between microvascular complications in diabetic populations and severe bone abnormalities [36]. The authors of the Chronic Renal Insufficiency Cohort study, who analyzed risk factors for CVD and renal failure progression, demonstrated that the coexistence of DM among patients with CKD was independently related to higher levels of phosphate, PTH, and FGF23 compared with nondiabetic individuals [37]. In contrast, according to Spanish researchers, the FGF23 levels were comparable in diabetic versus nondiabetic patients [38]. Different results may be explained by the fact that Túñon et al. enrolled subjects with better kidney function in their study. An intriguing study was conducted by Yeung and colleagues [39]. They evaluated the FGF23 level in T2DM patients with eGFR > 60 mL/min/1.73 m^2^, and showed that FGF23 was significantly higher in this diabetic population. In our study, we obtained consistent results; however, it has not been clarified what conditions determine FGF23 levels in patients with DM and preserved kidney function. There are several hypotheses elucidating this phenomenon. (1) Glycerol-3-phosphate (G3P) is a newly discovered kidney-derived metabolite that induces FGF23 production during renal failure [40]. However, dysregulated metabolism of G3P is typical for DM irrespective of kidney function [41]. Hence, G3P/FGF23 may be a novel axis in DM. (2) Decreased bone formation rates are typical for diabetic populations, and are associated with the elevated secretion of FGF23 [42]. (3) Early tubular injury in DM precedes the decline in eGFR or albuminuria, and contributes to a rise in FGF23 level [37,43]. (4) Hyperglycemia leads to formation of advanced glycation end products, which cause an increase in the FGF23 level [44,45]. (5) Patients with T2DM, especially the obese, are said to be in the proinflammatory state, and inflammation is widely known as a major trigger of FGF23 increase [46,47].

Sodium-glucose cotransporter 2 (SGLT-2) inhibitors are a group of relatively new antidiabetic drugs. Initially, their discovery caused considerable excitement among diabetologists; however, shortly afterwards the use of SGLT-2 inhibitors appeared to be associated with favorable outcomes in kidney disease and HF as well, irrespective of DM. Large clinical trials that investigated the role of empagliflozin, canagliflozin, and dapagliflozin showed impressive benefits from SGLT-2 inhibition in patients with high CV risk [48,49,50,51,52]. Several studies demonstrated that SGLT-2 inhibitor treatment increased phosphate, PTH, and FGF23 levels [53,54]. Perhaps phosphate and glucose transporters use the same sodium gradient, and may limit each other [55]. Insulin, which was discovered as a treatment for DM 100 years ago, has also gained interest regarding FGF23 concentration. Data concerning this relationship are rather poor. A group of researchers conducted a study indicating that insulin may contribute to the reduction in FGF23 levels in humans and mice [56]. In our study, we found no correlation between any medications for DM. To our knowledge, no studies have so far explored the association between FGF23 and biguanide or sulfonylureas treatment.

According to the current state of the art and our results, the role of FGF23 as a biomarker of CV risk among patients with IHD is limited, especially when MI is the only manifestation of IHD. The question therefore arises regarding whether diverse clinical syndromes comprising IHD should not be evaluated separately with respect to the FGF23 level. Multiple publications have indicated that FGF23 may perhaps be used effectively as a tool for CV risk stratification among subjects exhibiting HF. Furthermore, our study demonstrated that DM might be associated with increased levels of FGF23, regardless of IHD and CKD. Supposedly, it may predict the development of diabetic nephropathy in the future or the incidence of CV events in an extended follow-up. Moreover, the present study showed no evidence regarding the relationship between the FGF23 level and medications for DM. Data from clinical trials are eagerly awaited to further assess the role of FGF23 in DM, IHD, and HF. Evaluation of the antidiabetic drugs’ value in FGF23 level reduction is also highly desirable.

## 4. Study Limitations

Our study had certain limitations. There were multiple conditions that may have affected the FGF23 level. Furthermore, the sample size was relatively small, but in spite of everything, to our knowledge there are few studies that investigated the FGF23 correlations with IHD or glucose level among patients with long-standing T2DM, good glycemic control, and preserved renal function. Moreover, FGF23 measurement may have been associated with technical problems. Finally, the widespread usefulness of FGF23 was limited, since its normal range has not been established yet.

## 5. Materials and Methods

A database providing information on diabetics attending the Diabetes Outpatient Clinic in Lodz (Poland) between 2019 and 2021 was used for this study. The registry enrolled 6424 patients with DM, of whom 102 met the following inclusion criteria for the present research: (1) age > 18 years; (2) T2DM duration of >10 years; (3) regular follow-up care by a mutual diabetologist; and (4) HbA1c ≤ 8%. Individuals with any of the following conditions were excluded: (1) active infection; (2) malignant tumor; or (3) missing key clinical data. Participants were assigned to one of two groups based on the occurrence of IHD: Group 1 (non-IHD, n = 68) or Group 2 (IHD, n = 34).

Demographic and clinical characteristics were gathered during a face-to-face or telephone interview, and involved, among others: sex, age, body weight, height, BMI, T2DM duration, smoking status, medication usage, fasting glucose level, and family history of heart disease or diabetes. The patient interview included medical history, and focused primarily on CVDs (HA, IHD, MI), arrythmias, and other related diseases.

Our study retrospectively analyzed a dataset of routine clinical laboratory tests ordered by the diabetologist. The data table encompasses the following parameters: HbA1c (%), creatinine (Cr; mg/dL), eGFR (mL/min/1.73 m^2^), TC (mg/dL), HDL-C (mg/dL), non-HDL-C (mg/dL), LDL-C (mg/dL), and TG (mg/dL).

The FGF23 levels in samples were collected during previously scheduled visits to the Diabetes Outpatient Clinic. All the samples were obtained in the morning, after an overnight fast lasting at least 10 h, and stored at −80 °C before analysis. Serum intact FGF23 (iFGF23) levels were determined using a sandwich enzyme-linked immunosorbent assay (ELISA) with a FGF23 ELISA kit (SunRed Biological Technology, Shanghai, China). In healthy adults, the reference limits for serum iFGF23 concentration are 8.2–54.3 pg/mL [15].

All patients underwent echocardiograms and electrocardiograms at the Department of Interventional Cardiology in Lodz (Poland). The tests were separately reviewed and analyzed by two independent cardiologists.

Prevalent T2DM and IHD were defined at baseline of the study by self-report and subsequent validation by clinical records or medical history. CKD was defined as decreased kidney function characterized by eGFR less than 60 mL/min/1.73 m^2^. eGFR was calculated using the MDRD formula. Fasting and postprandial blood glucose were measured using finger-tip blood samples after an overnight fast lasting at least 8 h and following meals by 2 h, respectively. Arterial hypertension was defined as: (1) treatment with antihypertensive drugs; (2) previous diagnosis; and (3) systolic blood pressure of ≥140 mmHg and/or diastolic blood pressure of ≥90 mmHg on two occasions. BMI was calculated by dividing body weight in kilograms by the square of the body length in meters (kg/m^2^). Overweight was defined as a BMI between 25.0 and 29.9 kg/m^2^, and obese as a BMI ≥ 30 kg/m^2^.

All statistical analyses were performed using STATISTICA v. 13 software (StatSoft Polska, Kraków, Poland). For all tests, we used *p* = 0.05 as the threshold of statistical significance. The Shapiro–Wilk normality test was used to verify the distribution assumptions for normality. Categorical variables are represented as the number of observations (N) and the corresponding percentages (%). Quantitative variables are presented as median and IQR. Pearson’s χ2 test was used to check group equality. If the number of cases was less than 5, Yates’s correction for continuity was used. Continuous variables were analyzed with a nonparametric test. In order to compare two independent trials, the Mann–Whitney U test was used. Correlations were assessed by using Spearman’s rank correlation coefficient.

The study protocol was approved by the Ethics Committee of the District Medical Chamber in Lodz in accordance with the principles of the Declaration of Helsinki. Written informed consent was obtained from all patients before participating in the study.

## 6. Conclusions

FGF23 has gained wide attention in various fields of medicine, especially in nephrology. However, this protein remains mysterious due to its wide influence on multiple metabolites in the human body. FGF23 has become a part of the bone–kidney axis that influences vitamin D metabolism. It plays a pivotal role in calcium phosphate metabolism regulation. Many publications underline also that FGF23 participates in interorgan signaling. This study demonstrated that the FGF23 level was increased in all patients with long-standing T2DM, good glycemic control, and relatively preserved renal function. At the same time, we did not find simple correlations between FGF23 and glucose levels. Our analysis also unambiguously indicated that the role of FGF23 as a CV risk factor should not be overestimated among these patients, regardless of IHD status. Further larger studies are necessary to confirm our findings. Furthermore, in future investigations, it would be valuable to assess the pathophysiological cardiac effects of FGF23 and elucidate whether FGF23 secretion causes heart disease or vice versa.

## Figures and Tables

**Table 1:** Baseline characteristics.

Variable	Total *N* = 102	Without IHD *N* = 68	With IHD *N* = 34	*p* Value
Age, years	69 (66–74)	69 (66–74)	70 (68–74)	0.420
Female sex	43 (42)	34 (33)	9 (9)	0.024
T2DM duration, years	19 (14–25)	18 (13–24)	22 (16–27)	0.182
Fasting blood glucose, mg/dL	120 (110–130)	120 (110–130)	130 (110–140)	0.396
Postprandial blood glucose, mg/dL	150 (130–170)	140 (120–170)	150 (140–160)	0.238
BMI, kg/m^2^	28 (25–32)	29 (25–33)	27 (26–32)	0.337
Arterial hypertension	88 (86)	54 (53)	34 (33)	0.044
Myocardial infarction	29 (28)	0	29 (28)	0.000
CABG	9 (9)	0	9 (9)	0.000
Stroke	9 (9)	5 (5)	4 (4)	0.711
Atrial fibrillation	11 (11)	3 (3)	8 (8)	0.094
Diabetic retinopathy	20 (20)	10 (10)	10 (10)	0.077
Diabetic neuropathy	12 (12)	7 (7)	5 (5)	0.514
Diabetic foot syndrome	5 (5)	2 (2)	3 (3)	0.417
Family history of heart disease	51 (50)	34 (33)	17 (17)	1.0
Family history of diabetes mellitus	64 (63)	44 (43)	20 (20)	0.562
Current and former smokers	75 (74)	46 (45)	29 (29)	0.095
Non-smokers	27 (26)	22 (21)	5 (5)	0.095

Note: Data are expressed as median (interquartile range) or number (%). Abbreviations: IHD, ischemic heart disease; T2DM, type 2 diabetes mellitus; BMI, body mass index; CABG, coronary artery bypass graft surgery.

**Table 2:** Laboratory test results.

Variable	Total N = 102	Without IHD N = 68	With IHD N = 34	*p*-Value
FGF23, pg/mL	253 (218–531)	258 (212–548)	253 (222–531)	0.861
Cr, mg/dL	0.86 (0.75–1.0)	0.815 (0.73–0.95)	0.93 (0.84–1.27)	0.002
eGFR, mL/min/1.73 m^2^	80 (70–94)	84 (74–95)	79 (55–90)	0.035
HbA1c, %	6.85 (6.5–7.7)	6.85 (6.35–7.5)	6.85 (6.5–7.8)	0.422
TC, mg/dL	152 (125–186)	157 (128–194)	134 (124–178)	0.226
HDL-C, mg/dL	49 (42–59)	51 (43–62)	44 (41–53)	0.029
LDL-C, mg/dL	72 (51–98)	76 (53–100)	66 (48–98)	0.424
non-HDL-C, mg/dL	96 (75–126)	104 (75–126)	91 (78–127)	0.677
TG, mg/dL	126 (94–169)	122 (89–169)	131 (105–173)	0.244

Note: Data are expressed as median (interquartile range). Abbreviations: IHD, ischemic heart disease; FGF23, fibroblast growth factor 23; Cr, creatinine; eGFR, estimated glomerular filtration rate; HbA1c, hemoglobin A1c; TC, total cholesterol; HDL-C, high-density lipoprotein cholesterol; LDL-C, low-density lipoprotein cholesterol; TG, triglycerides.

**Table 3:** Transthoracic echocardiographic parameters.

Variable	Total *N* = 102	Without IHD *N* = 68	With IHD *N* = 34	*p*-Value
LVEF, %	56 (52–60)	56 (54–61)	55 (52–60)	0.038
LVESV, mL	32 (29–36)	31 (28–35)	36 (29–39)	0.007
LVEDV, mL	46 (43–50)	45 (43–48)	48 (45–53)	0.009
LVMI, g/m^2^	101 (84–115)	97 (83–108)	113 (91–131)	0.004
LA volume, mL	56 (44–75)	50 (42–63)	68 (56–83)	0.0002
LAVI, mL/m^2^	29 (23–37)	26 (22–33)	33 (30–42)	0.0003
TAPSE, mm	22 (19–24)	22 (20–25)	20 (18–23)	0.008
RVOT proximal diameter, mm	33 (30–35)	32 (30–34)	34 (30–36)	0.067
IVSs, mm	16 (15–17)	16 (15–17)	17 (14–18)	0.342
IVSd, mm	11 (10–13)	11 (10–12)	12 (11–14)	0.098

Note: Data are expressed as median (interquartile range). Abbreviations: IHD, ischemic heart disease; LVEF, left ventricular ejection fraction; LVESV, left ventricular end systolic volume; LVEDV, left ventricular end diastolic volume; LVMI, left ventricular mass index; LA, left atrium; LAVI, left atrial volume index; TAPSE, tricuspid annular plane systolic excursion; RVOT, right ventricular outflow tract; IVSs, interventricular septum thickness at end-systole; IVSd, interventricular septum thickness at end-diastole.

**Table 4:** FGF23 concentration versus use of medications for diabetes mellitus.

Class	Patients Taking Medication	FGF23 Concentration		*p* Value
		Taking Medication	Not Taking Medication	
Biguanide	83 (82)	253 (218–529)	356 (212–713)	0.525
Sulphonylureas	28 (28)	260 (224–669)	253 (214–479)	0.474
Thiazolidinediones	4 (4)	291 (223–590)	253 (218–531)	0.743
α-glucosidase inhibitors	12 (12)	315 (231–659)	253 (214–487)	0.520
SGLT-2 inhibitors	24 (24)	256 (204–364)	253 (222–655)	0.280
DPP-4 inhibitors	13 (13)	487 (268–727)	252 (214–467)	0.086
GLP-1 agonists	1 (1)	867 (867–867)	253 (218–529)	1.000
Insulins	47 (46)	268 (222–531)	252 (206–656)	0.535

Note: Data are expressed as number (%) or median (interquartile range). Abbreviations: IHD, ischemic heart disease; SGLT2, sodium-glucose cotransporter 2; DPP-4, dipeptidyl peptidase 4; GLP-1, glucagon-like peptide 1.

## Data Availability

The data presented in this study are available in the main article and the Supplementary Materials.

## Supplementary Materials

The following supporting information can be downloaded at: https://www.mdpi.com/2218-1989/12/6/498/s1, Table S1: Correlations between FGF23 and other variables; Table S2: Correlations between various variables and overt IHD.
