# Supplementary material for: Fibroblast Growth Factor 23 and Cardiovascular Risk in Diabetes Patients—Cardiologists Be Aware
Source: Metabolites. 2022 May 30;12(6):498. doi: 10.3390/metabo12060498 (PMC9254740; doi:10.3390/metabo12060498)
Supplement: Supplementary file 1 [file metabolites-12-00498-s001.zip › metabolites-1714181-supplementary.pdf]

Table S1. Correlations between FGF23 and other variables

Variable	Spearman's correlation coefficient	
	**r**	***p***
Cr, mg/dL	-0.0577	0.571
eGFR, ml/min/1,73m^2	-0.0196	0.848
HbA1c, %	-0.1456	0.151
TC, mg/dL	0.0377	0.711
HDL-C, mg/dL	0.1569	0.121
LDL-C, mg/dL	-0.0027	0.979
non-HDL-C, mg/dL	-0.0241	0.813
TG, mg/dL	-0.0714	0.483
LVEF, %	0.0873	0.393
LVESV, mL	-0.1514	0.137
LVEDV, mL	-0.1328	0.193
LVMI, g/m^2	-0.0842	0.410
LA volume, mL	-0.0624	0.542
LAVI, mL/m^2	0.0011	0.992
TAPSE, mm	0.0881	0.388
RVOT proximal diameter, mm	-0.1511	0.137
IVSs, mm	0.0127	0.901
IVSd, mm	0.0369	0.719

Note: Values are expressed as r (Spearman's correlation coefficient) and *p* values.
Abbreviations: FGF23, fibroblast growth factor 23; Cr, creatinine; eGFR, estimated glomerular filtration rate; HbA1c, hemoglobin A1c; TC, total cholesterol; HDL-C, high-density lipoprotein cholesterol; LDL-C, low-density lipoprotein cholesterol; TG, triglycerides; LVEF, left ventricular ejection fraction; LVESV, left ventricular end systolic volume; LVEDV, left ventricular end diastolic volume; LVMI, left ventricular mass index; LA, left atrium; LAVI, left atrial volume index; TAPSE, tricuspid annular plane systolic excursion; RVOT, right ventricular outflow tract; IVSs, interventricular septum thickness at end-systole; IVSd, interventricular septum thickness at end-diastole.

Table S2. Correlations between various variables and overt IHD.

Variable	Spearman's correlation coefficient	
	r	*p*
Cr, mg/dL	0.3514	0.000
eGFR, ml/min/1,73m^2	-0.2577	0.010
HbA1c, %	0.1032	0.309
TC, mg/dL	-0.1348	0.184
HDL-C, mg/dL	-0.2260	0.024
LDL-C, mg/dL	-0.1018	0.316
non-HDL-C, mg/dL	-0.553	0.587
TG, mg/dL	0.1332	0.189
LVEF, %	-0.3078	0.002
LVESV, mL	0.3006	0.003
LVEDV, mL	0.3067	0.002
LVMI, g/m^2	0.3221	0.001
LA volume, mL	0.3227	0.001
LAVI, mL/m^2	0.3053	0.002
TAPSE, mm	-0.2459	0.015
RVOT proximal diameter, mm	0.1245	0.222
IVSs, mm	0.0680	0.506
IVSd, mm	0.1706	0.093

Note: Data are expressed as r (Spearman's correlation coefficient) and *p* values.
Abbreviations: IHD, ischemic heart disease; Cr, creatinine; eGFR, estimated glomerular filtration rate; HbA1c, hemoglobin A1c; TC, total cholesterol; HDL-C, high-density lipoprotein cholesterol; LDL-C, low-density lipoprotein cholesterol; TG, triglycerides; LVEF, left ventricular ejection fraction; LVESV, left ventricular end systolic volume; LVEDV, left ventricular end diastolic volume; LVMI, left ventricular mass index; LA, left atrium; LAVI, left atrial volume index; TAPSE, tricuspid annular plane systolic excursion; RVOT, right ventricular outflow tract; IVSs, interventricular septum thickness at end-systole; IVSd, interventricular septum thickness at end-diastole.
